# Best practices for the management of febrile seizures in children

**DOI:** 10.1186/s13052-024-01666-1

**Published:** 2024-05-12

**Authors:** Alessandro Ferretti, Antonella Riva, Alice Fabrizio, Oliviero Bruni, Giuseppe Capovilla, Thomas Foiadelli, Alessandro Orsini, Umberto Raucci, Antonino Romeo, Pasquale Striano, Pasquale Parisi

**Affiliations:** 1 Pediatrics Unit, Neurosciences, Mental Health and Sensory Organ (NESMOS) Department, Faculty of Medicine and Psychology, S. Andrea Hospital, Sapienza University, via di Grottarossa 1035/1039, Rome, 00189 Italy; 2 IRCCS Giannina Gaslini, Genoa, Italy; 3 Department of Neurosciences, Rehabilitation, Ophthalmology, Genetics, Maternal and Child Health, University of Genoa, Genoa, Italy; 4 Department of Social and Developmental Psychology, S. Andrea Hospital, Sapienza University, Rome, Italy; 5 Child Neuropsychiatry Department, Epilepsy Center, Mantova, Italy; 6 C. Poma HospitalFondazione Poliambulanza, Brescia, Italy; 7 Pediatric Clinic, Fondazione IRCCS Policlinico San Matteo, Pavia, Italy; 8 Pediatric Neurology, Pediatric University Department, Azienda Ospedaliera Universitaria Pisana, University of Pisa, Pisa, Italy; 9 General and Emergency Department, Bambino Gesù Children’s Hospital, IRCCS, Rome, Italy; 10 Fatebenefratelli Hospital, ASST Fatebenefratelli-Sacco, Milan, Italy

**Keywords:** Febrile seizure, Children, Management, Prognostic factors, Red flags, Recommendations for caregivers

## Abstract

Febrile seizures (FS) are commonly perceived by healthcare professionals as a self-limited condition with a generally ‘benign’ nature. Nonetheless, they frequently lead to pediatric consultations, and their management can vary depending on the clinical context. For parents and caregivers, witnessing a seizure can be a distressing experience, significantly impacting their quality of life. In this review, we offer an in-depth exploration of FS management, therapeutic interventions, and prognostic factors, with the aim of providing support for physicians and enhancing communication with families. We conducted a comprehensive literature search using the PubMed and Web of Science databases, spanning the past 50 years. The search terms utilized included “febrile seizure,” “complex febrile seizure,” “simple febrile seizure,” in conjunction with “children” or “infant.” Only studies published in English or those presenting evidence-based data were included in our assessment. Additionally, we conducted a cross-reference search to identify any additional relevant data sources. Our thorough literature search resulted in a compilation of references, with carefully selected papers thoughtfully integrated into this review.

## Introduction

Febrile seizures (FS) are commonly perceived by healthcare professionals as a self-limited condition with a generally ‘benign’ nature. Nonetheless, they frequently lead to pediatric consultations, and their management can vary depending on the clinical context [1]. For parents and caregivers, witnessing a seizure can be a distressing experience, significantly impacting their quality of life [2].

In this review, we offer an in-depth exploration of FS management, therapeutic interventions, and prognostic factors, with the aim of providing support for physicians and enhancing communication with families. We conducted a comprehensive literature search using the PubMed and Web of Science databases, spanning the past 50 years. The search terms utilized included “febrile seizure,” “complex febrile seizure,” “simple febrile seizure,” in conjunction with “children” or “infant.” Only studies published in English or those presenting evidence-based data were included in our assessment. Additionally, we conducted a cross-reference search to identify any additional relevant data sources. Our thorough literature search resulted in a compilation of references, with carefully selected papers thoughtfully integrated into this review.

## What are febrile seizures?

FS are “provoked” epileptic seizures starting during a febrile event/episode that occur in the absence of infection in the central nervous system (CNS), typically affecting children aged 6 months to 5 years [3, 4]. Although categorized as epileptic seizures, in the majority of cases they do not lead to a diagnosis of epilepsy [5, 6].

FS affect approximately 2–5% of children in the United States and Western Europe [7, 8] and 6 to 9% among Japanese ones [9]. The peak incidence of the first FS typically occurs during the second year of a child’s life [7]. The precise causes of FS remain not entirely clear. A distinctive vulnerability of the developing brain to fever and relatively minor viral illnesses within a specific developmental window, resulting in seizures, in only a subset of children, prompts the question of why these children experienced seizures while others did not. Factors statistically correlated with FS encompass a family history of such seizures, indications of neurological dysfunction or developmental disabilities, delayed neonatal discharge, and attendance at day care [10].

The prevailing etiopathogenic hypothesis is that FS has a notable genetic predisposition Polygenic inheritance has been suggested, although an autosomal dominant inheritance pattern of a defined “FS susceptibility trait” has been identified in a few families [11, 12]. If a child experiences FS, the risk that their sibling will also experience one range from 10 to 45% [13]. Monozygotic twins exhibit higher concordance rates for FS compared to dizygotic twins (53% versus 18%) [14]. Notably, compelling evidence has emerged from linkage studies, reporting linkages on multiple chromosomes such as 2q [15], 5q [16], 8q [17], 19p [18], and 19q [19], with the most robust linkage on chromosome 2q and specifically to genes responsible for sodium channel receptors. Another significant syndrome associated with FS is genetic epilepsy with febrile seizures plus (GEFS+). GEFS + has been coined to identify a syndrome characterized by the onset of FS typically between 6 months and 6 years of age, marked by the presence of FS that may persist beyond the usual resolution age or be accompanied by afebrile seizures, which can be generalized or focal [20]. While a genetic predisposition is evidently insufficient on its own to trigger FS, fever is a requisite, and up to 82% of FS occur during viral infections [21]. The specific type of viral infection is not predictive of complex features or future recurrences [22]. Nonetheless, viruses most frequently associated with FS include human herpesvirus 6, influenza, adenovirus, respiratory syncytial virus, parainfluenza and severe acute respiratory syndrome coronavirus 2 (SARS-CoV-2) [22–25]. Viral infections can trigger an inflammatory state that may facilitate the occurrence of febrile seizures [26, 27]. It remains unclear if there is a specific fever threshold at which a febrile seizure can occur [28], with some studies indicating 38 °C and others 38.4 °C [29, 30]. Likewise, data to support a rapid temperature increase being more significant than the peak temperature attained is lacking [29, 31, 32]. The occurrence of epileptic seizures in the context of fever before the age of 6 months should raise suspicion of the onset of epilepsy with a genetic etiology, such as variants in the *SCN1A* [33] or PCDH19 [34] genes. FS can occur in older children, albeit very rarely after the age of 6 years [35].

## “Simple” or “complex” FS

**Fig. 1:**
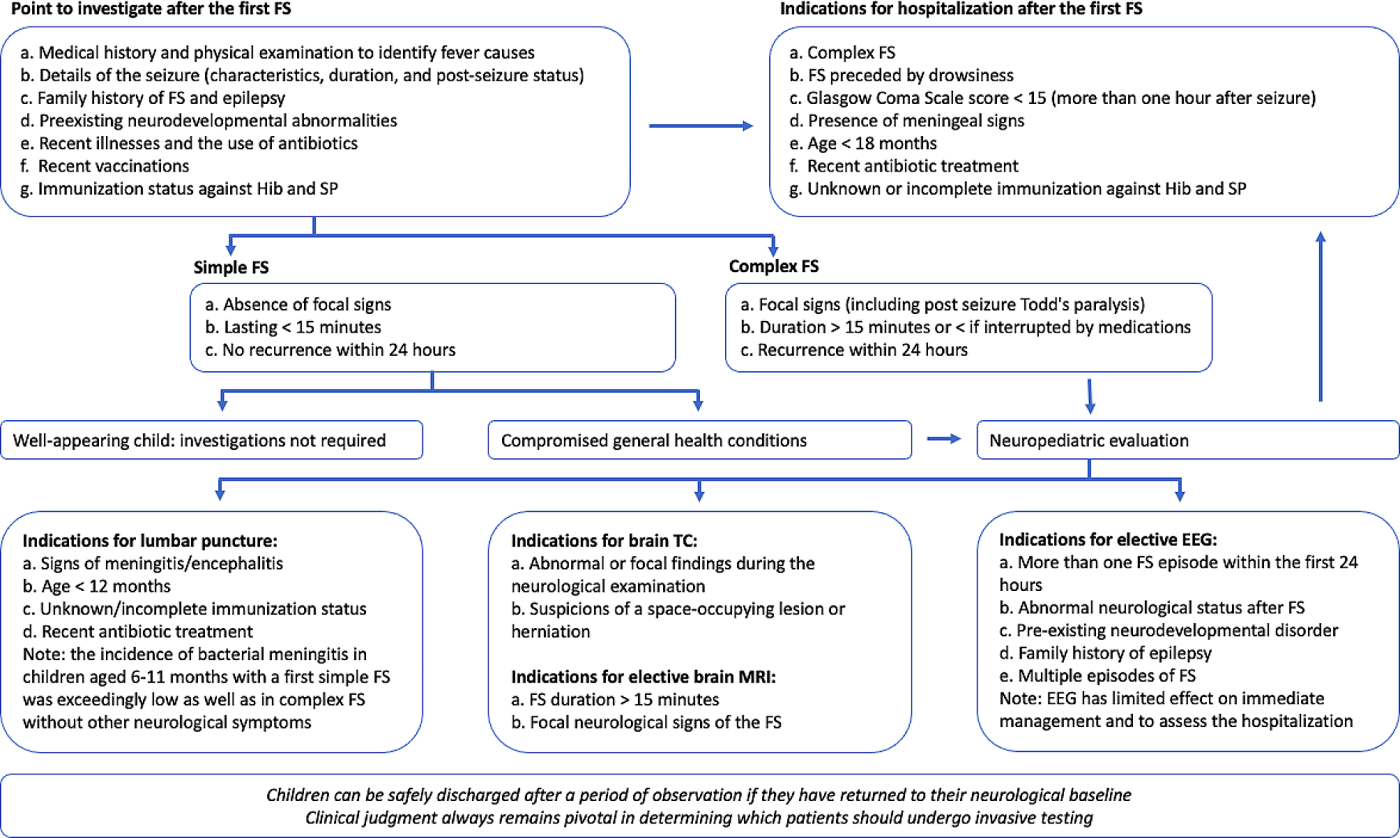
Management of children with first febrile seizure. FS = febrile seizure; Hib = Haemophilus influenzae type b; SP = Streptococcus pneumoniae

Classically, FS are categorized as either “simple” or “complex” based on the presence of focal signs, duration, and recurrence within a single infectious episode (Fig. 1) [36]. Approximately 20–35% of FS are classified as complex [37, 38], and their prevalence increases to up to 45% in children under 12 months of age [39].

The definition provided by the American Academy of Pediatrics explicitly excludes children with neurological disorders predisposing to later seizures (e.g., cerebral palsy) [3, 4]. This is not explicitly specified in the definition provided by the International League Against Epilepsy, although it might be suggested by the exclusion of acute symptomatic seizures [40]. Neither of the above definitions explicitly excludes children with pre-existing neurodevelopmental disorders such as autism spectrum disorders, even though they may experience febrile seizures [41].

A prolonged (> 5 min) FS may eventually result in a febrile status epilepticus (FSE). The definition of FSE traditionally involved at least 30 min of continuous seizure activity or 30 min of recurrent seizures without complete recovery of consciousness in between. FSE accounts for 25–52% of all cases of status epilepticus in children, although it constitutes a small portion of FS incidents [42, 43]. The recurrence rate for FSE within one year after the first FSE episode is 16% [44].

## Management

Prehospital and emergent management should prioritize stabilizing the child by addressing the ABCs (airway, breathing, and circulation). The majority of FS are self-limiting and tend to resolve before children arrive at the hospital. However, it has been demonstrated that prolonged FS are unlikely to spontaneously terminate [45]. Consequently, seizures lasting longer than five minutes are improbable to cease on their own, and the administration of a benzodiazepine (BDZ) is recommended to terminate the seizure [46]. The ideal BDZ to be used in an early phase of seizure control should have an easy and socially acceptable route of administration, a rapid onset but at the same time limited adverse events (in terms of respiratory depression) [47]. With this in mind, buccal midazolam (MDZ) and rectal diazepam (DZP) are the first choice as rescue therapy [47]. Particularly, MDZ has favorable pharmacokinetic properties that ensure rapid action and a short half-life, supporting its use with various administration routes (intravenous, intramuscular, buccal, intranasal). In Italy, buccal MDZ has received approval for the treatment of prolonged febrile seizures in children aged above three years and should be administered at a dose of 0.5 mg/kg with pre-dosed syringes formulations (3–4 years: 5 mg; 5–9 years: 7.5 mg; 10–18 years: 10 mg) [48–50]. Rectal DZP has limitations due to the variable and unpredictable rectal absorption. Moreover, it has a higher risk of respiratory depression than MDZ [50]. The recommendation is to prefer rectal DZP for children aged less than 3 years (at a dose of 5 mg) with a subsequent switch to buccal MDZ. After a first dose of BDZ, a second one could be administered after 5 min if the FS has not stopped. Administering more than two doses of benzodiazepines is not recommended due to the potential risk of inducing respiratory depression [49]. If intravenous access is available, other BDZs could be considered. For example, a Cochrane review published in 2018 concluded that intravenous lorazepam (0.1 mg/kg/dose; max 4 mg/dose) and diazepam (0.2 mg/kg/dose; max 10 mg) have similar rates of seizure cessation and potential respiratory depression. Another option is intravenous midazolam (0.2 mg/kg/dose; max 10 mg). Management of FS is summarized in Fig. 1. The acute management of FSE follows the established protocol for managing status epilepticus of any cause [51] and is not the focus of this review.

## Diagnostic assessments

The evaluation of a child with FS should begin with a medical history and a physical examination to determine the underlying cause of the fever. For FS, it is advisable to investigate how the episode occurred, its duration, and whether there is a history of other FS, epilepsy, or other brain disorders in the family. Additionally, it is necessary to consider recent illnesses, ongoing antibiotic use, recent vaccinations, and the child’s immunization status against Haemophilus influenzae type b and Streptococcus pneumoniae.

For children experiencing simple FS who are well-appearing, routine tests such as blood examinations, neuroimaging, or EEG are generally not required unless there is a clear need to ascertain the cause of the fever [4]. If a child has complex FS or experiences a simple FS accompanied by poor overall condition, it is recommended to undergo a comprehensive evaluation by a neuro-pediatrician. Additional tests are determined based on the child’s medical history and their presentation during this examination.

In those cases, a FS raises the concern for meningitis and a neuro-pediatric examination is crucial in deciding whether to perform invasive tests like a lumbar puncture. If the child is older than one year and he/she is well-appearing, a lumbar puncture may not be necessary [4]. For infants under one year of age, there is a sense that the physical signs of meningitis might be more subtle, so making a lumbar puncture is strongly recommended. Particularly, for infants aged 6 to 12 months presenting with a seizure and fever, a lumbar puncture should be considered if the child is not adequately immunized against Haemophilus influenzae type b (Hib) or Streptococcus pneumoniae, or when the immunization status cannot be determined due to an increased risk of bacterial meningitis [4]. A lumbar puncture is also an option for children who have been pre-treated with antibiotics, as antibiotic treatment can mask the signs and symptoms of meningitis while still being insufficient to eradicate it [4]. Despite these guidelines, some authors have reported that experienced physicians rarely perform lumbar punctures [52]. Guedj et al. estimated that the risk of bacterial meningitis in children aged 6–11 months with a first simple FS was extremely low [53, 54]. Several studies highlight that bacterial meningitis is unlikely in children with complex FS without other neurological symptoms, particularly if the child is well-appearing [55, 56]. Clinical judgment always remains pivotal in determining which children should undergo invasive testing [57]. Similarly, the clinical history and neurological examination can assist in deciding whether neuroimaging is necessary for children with complex FS. In this regard, neuroimaging is generally not required for complex FS unless the child exhibits abnormal or focal findings during the neurological examination. If a child recovers promptly from FS, a head computed tomography (CT) scan is of limited value [58]. Notably, there are very few instances where children with complex FS show intracranial pathology in the absence of other signs or symptoms [59]. Brain CT scans are typically necessary when considering a lumbar puncture or if there are suspicions of a space-occupying lesion or herniation. However, the likelihood of identifying a lesion on neuroimaging that requires immediate neurosurgical or medical intervention is extremely low, making such investigations unnecessary for most children with complex FS [60].

When contemplating the execution of an electroencephalogram (EEG) following a FS, the guideline from the American Academy of Pediatrics specifies that an EEG should not be conducted in the assessment of a neurologically healthy child with a simple FS; this is because there is no evidence suggesting that EEG abnormalities can predict the recurrence of FS or the onset of epilepsy [4]. In case of complex FS, opinions are not unanimous. While some studies have demonstrated that an epileptiform EEG was not a sensitive measure and had a poor positive predictive value for the development of epilepsy among neurologically healthy or mildly delayed children with a first complex FS [61, 62], others have found that epileptiform discharges on EEGs are predictive risk factors for the development of epilepsy [63, 64]. A recent Cochrane review did not find any randomized controlled trials (RCTs) as evidence to support or refute the use of EEG and its timing after complex FS among children [65].

An EEG should be performed on a child presenting with a complex FS accompanied by abnormal neurological and developmental status, as the highest risk of epilepsy exists in this population [66]. Additionally, EEG plays a crucial role in supporting the diagnostic suspicion of herpes simplex encephalitis, the most prevalent form of sporadic encephalitis worldwide, in children with suggestive clinical manifestations [67, 68]. The diagnostic assessment of FS is summarized in Fig. 1.

## When is hospitalization recommended?

Hospitalization is often carried out for observation after the occurrence of a first FS [69, 70]. One of the primary reasons for observation is the potential for infections affecting the CNS and the concern about further seizures in the immediate aftermath. Factors that indicate a child’s admission for hospitalization include being drowsy before the seizure, having a Glasgow Coma Scale (GCS) score less than 15 more than an hour after the seizure, exhibiting signs of meningeal involvement, being under 18 months of age, having received antibiotic treatment before the FS, and having incomplete immune status [71]. Children with their first CFSs have a low risk of seizure recurrence during their hospital stay [72], and no predictors for seizure recurrence have been identified [73]. However, if multiple seizures occur within 24 h of presentation, there is a risk of early recurrence and may warrant admission. In any case, the Italian League Against Epilepsy [74], the Joint Working Group of the Research Unit of the Royal College of Physicians and the British Pediatric Association Commission [75], and the World Health Organization guidelines [71] all recommend routine admission for observation for all children presenting with complex FS. EEG has a limited impact on acute management and should not be used as a basis for admission [72]. The majority of children can be safely discharged after a period of observation if they have returned to their neurological baseline.

## Red flags

**Fig. 2:**
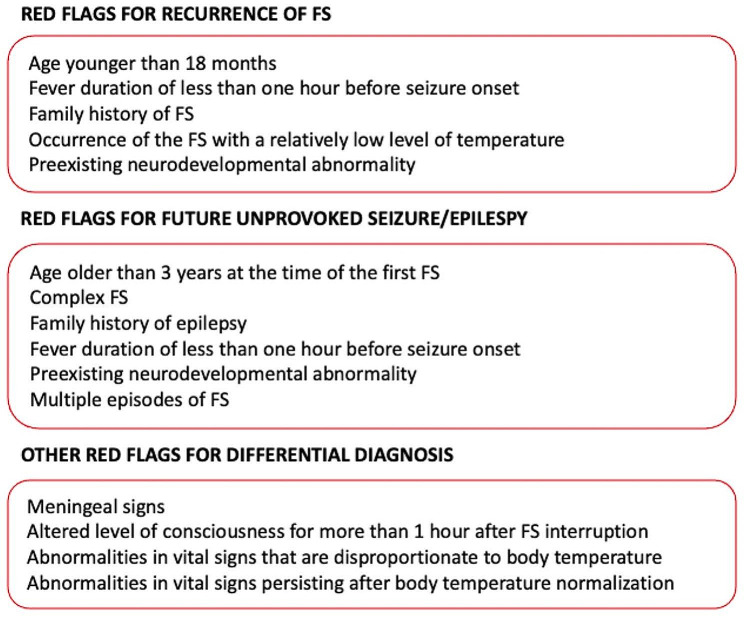
Red flags of febrile seizures

The comprehensive list of red flags can help physicians in assessing the risk of FS recurrence and future unprovoked seizures, as well as identify children who require more extensive emergency evaluations [76–80]. These red flags for each risk are summarized in Fig. 2. Approximately 30–50% of children who experience their first FS will have subsequent episodes of FS [8, 76, 80, 81].

One of the most extensively studied negative prognostic factors is a family history of FS [82]. Many studies have also suggested that an underlying brain disorder might increase the risk. Premature birth, delayed discharge from the neonatal intensive care unit, and developmental delay are potential indicators of suboptimal brain function, although there is conflicting evidence definitively linking these factors to FS [8, 10, 83, 84].

Recurrences seem to be more likely in children whose initial FS occurred with a relatively low fever and a short duration between the onset of fever and FS [79, 82, 85]. Identifying independent factors, including a young age at onset, a history of FS in a first-degree relative, a low degree of fever at the emergency department, and a brief duration between the onset of fever and the initial seizure, has shown that children with all four of these factors have a recurrence risk for FS of 70%, whereas those with no factors have a recurrence risk of only 20% [79]. There is no difference in the risk of recurrence based on whether the initial FS was simple or complex [79].

Individuals with FS seizures have a risk of subsequent epilepsy of 1%, which is higher than that in the general population but not clinically significant [86]. Conversely, complex FS are followed by epilepsy in 4–15%, depending on the number of complex features [76, 81, 87]. From early observations, prior neurological and developmental status, and FS with complex features have been recognized as important predictors of epilepsy [76]. More recently, the main prognostic factors for the development of epilepsy after FS have been identified as complex FS, which increases the risk by 3.6 times, age at onset of FS beyond the third year of life, which raises the risk by 3.8 times, a positive family history of epilepsy, which increases the risk by 7.3 times, and multiple episodes of FS, which raises the risk by about 10 times. Focality at the first and second FS recurrence increases the risk of epilepsy by about 9.7 and 11.7 times, respectively [81]. Additionally, multivariate analysis has shown that maternal history of epilepsy is a strong prognostic factor [81], but this finding has not been replicated in subsequent studies. An epileptiform EEG was not a sensitive measure and had a poor positive predictive value for the development of epilepsy among neurologically healthy or mildly delayed children with a complex FS [62]. The recurrence rate for FSE within one year after the first FSE episode is 16% [44]. Finally, there are conflicting results regarding the development of subsequent FS or the onset of epilepsy after FSE in an otherwise normal child [42, 88].

## Prevention

The use of antipyretic medications may provide relief for a feverish child but it does not prevent FS [89]. Well-constructed randomized trials of appropriate doses of acetaminophen (10 mg/kg/dose four times per day) [90], ibuprofen (5 mg/kg/dose every 6 h) [91], and rectal diclofenac (1.5 mg/kg/dose every 6 h) [92] have failed to show any benefit in preventing FS. Consistently, a recent systematic review did not find a clear benefit of using antipyretics to prevent FS within the same fever episode and during distant fever episodes [93]. Another meta-analysis failed to identify benefits for children with FS from intermittent prophylaxis. Specifically, they found no significant benefit for intermittent phenobarbital, phenytoin, valproate, pyridoxine, ibuprofen, or zinc sulfate compared to placebo or no treatment; nor for diclofenac compared to placebo followed by ibuprofen, paracetamol, or placebo; nor for continuous phenobarbital compared to diazepam, intermittent rectal diazepam compared to intermittent valproate, or oral diazepam compared to clobazam [94]. However, reduced recurrence rates were seen for intermittent diazepam and continuous phenobarbital, with adverse effects in up to 30% of children [94].

When considering the use of chronic anti-seizure medication (ASM), studies have demonstrated the effectiveness of phenobarbital, primidone, and valproic acid in preventing the recurrence of simple FS; however, the side effects of each ASM outweighed the benefits [95, 96]. Carbamazepine and phenytoin are not effective in preventing recurrent FS [78, 95]. Levetiracetam [94] can be an effective ASM in preventing the recurrence of complex FS. However, chronic prophylactic ASM for both simple and complex FS is not routinely recommended [95]. Finally, parents/caregivers should avoid co-sleeping with children, as it may be dangerous for their children and does not prevent FS [89].

## Vaccinations in children with FS

FS are not a reason to avoid vaccinations. Seizures linked to vaccinations, classified as vaccine proximate seizures (VPSs), can manifest within a two-week period post-vaccination, regardless of the presence of fever. A retrospective study involving 119 children has shown that for those experiencing a solitary VPS without further seizure episodes, the likelihood of experiencing another VPS upon subsequent vaccination is rare [97]. Conversely, children who encountered multiple seizures not related to vaccination following their initial VPS episode (identified as VPS+) demonstrated a higher propensity for experiencing subsequent afebrile VPSs (42.6% compared to 15.5%, *P* = 0.002), were typically younger at the time of the first VPS occurrence (6.2 versus 12.5 months, *P* = 0.03), and had a greater chance of VPS recurrence following another vaccination, as compared to those with a single VPS event. For these particular cases, especially in children younger than 12 months, a thorough assessment and investigation for the diagnosis of Dravet syndrome is advised, along with taking extra precautions during revaccination due to their elevated risk of experiencing another VPS [97]. In relation to VPS, there can be instances of “afebrile benign convulsion” where the triggering event does not directly correlate with fever. Here, the significant factor is not the fever itself but the inflammation caused by the agent responsible for underlying inflammatory conditions, akin to the reaction seen in norovirus gastroenteritis [98]. Additionally, for these children with a slightly elevated incidence of FS observed within a 14-day period following vaccination [38, 99], this association is now understood to be primarily due to vaccine-induced fever in individuals who are genetically predisposed [100]. Vaccinations help prevent infections caused by common viruses or bacteria that can trigger FS, ultimately reducing the overall risk [30, 101]. Vaccines such as pneumococcal, meningococcal, and Haemophilus influenzae vaccines also play a crucial role in safeguarding children from encephalitis and meningitis, conditions that can lead to epileptic seizures. However, as mentioned at the beginning of this document, seizures related to CNS infections are not classified as FS.

The preventive impact of antipyretic medications on FS occurring after vaccination is not currently established. Nevertheless, it’s not generally recommended to administer antipyretic drugs routinely at the time of vaccination as they could potentially reduce the body’s immune response to several vaccine antigens [102].

## Prognosis

A key concern of children with FS is the possibility of long-term neurological sequelae. It is well demonstrated and accepted that short FS are not associated with an increased risk of neurological or cognitive impairments [77, 103–106].

This is more controversial for children with FSE [107–110]. Cognitive scores were similar within 6 weeks and at 1 year post FSE in a London study but a worse developmental outcome than controls has been observed [107]. The FEBSTAT study found similar scores initially between children with FSE when compared with children with simple FS, but lower scores in the FSE group after a year [108]. Long-term IQ findings (9 years post-FSE) were similar to short-term outcomes post-FSE in the same London cohort [110]. Several studies have chosen to examine FSE compared to brief FS to assess the risk of developing mesial temporal sclerosis (MTS) associated with temporal lobe epilepsy [111, 112]. These studies have concluded that the evidence for a causative relationship between MTS and FS is weak [113]. On the other hand, there is limited evidence to suggest that FSE can result in hippocampal abnormalities and subsequent adverse outcomes [111, 112]. Among the numerous etiologies of status epilepticus, the risk of developing epilepsy is lower after febrile SE [114].

## Mortality

A population-based cohort study found no increase in long-term mortality in children with simple FS compared with the general population. Children with complex FS (> 15 min or recurrence within 24 h) were more likely to die in the following two years when compared with children without FS (adjusted mortality rate ratio = 1.99), although this was at least in part secondary to pre-existing neurologic abnormalities and subsequent epilepsy [115]. The same article concludes that parents should be reassured that death after FS is very rare, even in high-risk children. Additionally, there does not appear to be any association between FS and sudden infant death syndrome [116]. This should be emphasized in discussions about FS with families [117]. However, this view has become rather controversial given recent studies that have identified an increased rate of FS in the largest cohort of sudden unexplained deaths in childhood (SUDC). Significantly increased rates of FS have also been observed among cases of sudden explained deaths in childhood (SEDC), primarily attributed to infections (mainly pneumonia and viral infections) and accidental deaths [118]. With these considerations, the possibility that FS might contribute to some SUDC and SEDC deaths is still not fully explainable [119]. Finally, the risk of death during hospitalization for the acute FSE episode and at 8 years and 6 months post-FSE is 0% [44, 120].

## Recommendations for caregivers

**Fig. 3:**
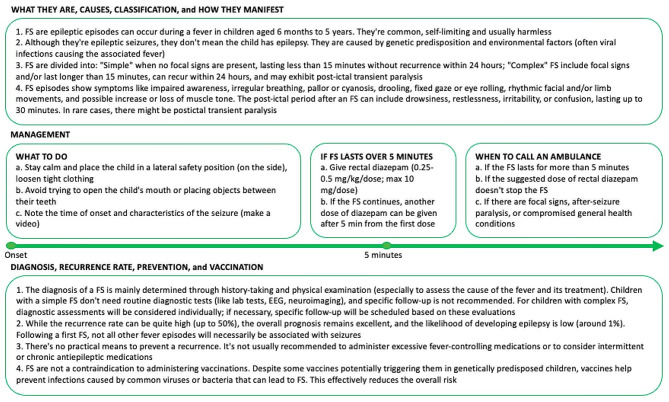
Recommendations for caregivers of children with febrile seizures

Special attention should be given to family counseling, and accurate information should be conveyed verbally and in writing. Parents whose child has experienced FS need to understand that preventing recurrence is not feasible. They should also be reassured that the issue will likely be resolved over the next few years without lasting effects. Additionally, it’s important to clarify that not all subsequent infectious or FS will necessarily trigger another seizure, thus minimizing “fever-phobia” [121]. Recently, a consensus was reached among child neurologists and pediatricians from five European countries regarding the information to be shared with families following FS [89]. Accordingly, we propose in Fig. 3a panel of recommendations to discuss with caregivers of children experiencing FS. The parental educational intervention program has demonstrated its effectiveness in enhancing parents’ limited knowledge, changing negative attitudes, reducing anxiety, and promoting better first-aid responses to FS [122].

## Discussion

FS are a common childhood condition, and while they have a relatively high recurrence rate, the overall prognosis is favorable, with a low risk of developing epilepsy. However, the distinction between simple and complex FS has been a point of emphasis in the medical community, and it’s worth noting that parents, often distressed during these episodes, may struggle to accurately recognize the features of the seizures. Such difficulties can lead to confusion, particularly when estimating seizure duration, which has been demonstrated to be inaccurate in witness descriptions of attacks. Such inaccuracies can potentially result in diagnostic errors and inappropriate treatment [123]. Recently, a retrospective study suggested reducing the cutoff for the duration of simple FS to 6 min, as they observed that the population with FS with a duration greater than 6 min presented EEG alterations at follow-up visits, neurological disorders, and a recurrence of FS during the following year [124]. However, an international consensus on this matter has not yet been reached, and currently, the cutoff duration between simple and complex FS remains at 15 min. The most reliable factor distinguishing between simple and complex seizures is the occurrence of repeated episodes within a 24-hour period. Nevertheless, differentiating between these two types of seizures is crucial as it guides the diagnostic path for the child and helps in avoiding unnecessary investigations. In cases of simple FS, additional assessments are not indicated. However, when dealing with complex seizures, it’s essential to acknowledge the uniqueness of each case, necessitating a considerate and comprehensive approach. In such situations, the decision of whether to proceed with an EEG, neuroimaging, or lumbar puncture should be carefully weighed, bearing in mind that CNS infection is the primary differential diagnosis. When no apparent risk factors are present, a prudent approach might involve outpatient EEG evaluation, especially if multiple complex features are evident. On the other hand, outpatient EEG should always be considered for children with multiple risk factors for epilepsy, such as developmental delay or a family history of epilepsy, particularly if they exhibit more than one defining feature of complex FS, due to the increased risk of subsequent nonfebrile seizures. The risk of epilepsy following FS depends on the type of seizure and the duration of follow-up. While the exact mechanisms linking FS to epilepsy are not fully understood, recent studies suggest a strong genetic link between FS and epilepsy [33, 125], warranting investigation in relevant cases. Consistent with existing literature, no ASM should be used to prevent recurrent FS, and fever control medications should not be administered beyond what is necessary to manage fever itself. Both intermittent and chronic ASMs are generally not recommended. Proper recognition and evaluation of red flags can guide appropriate management and interventions for affected children, establishing the foundation for suitable follow-up. Finally, detailed counseling with the child’s family is essential to improve the management of any new FS [89].

## Conclusions

In conclusion, FS are a common childhood condition with a relatively high recurrence rate. However, the overall prognosis is favorable, with a low risk of developing epilepsy. The distinction between simple and complex FS can be helpful in guiding the diagnostic assessment. Repeated episodes within a 24-hour period are the most reliable factor distinguishing between them. For simple FS, additional assessments are not indicated. In contrast, when dealing with complex FS, a comprehensive approach could be necessary. Decisions regarding EEG, neuroimaging, or lumbar puncture should be made carefully, considering the unique aspects of each case and the risk of CNS infection. Outpatient EEG evaluation is an option, especially for children with multiple risk factors for epilepsy. The risk of epilepsy following FS depends on the type of seizure and the duration of follow-up, with recent studies suggesting a genetic link between FS and epilepsy. It is important to note that no ASM should be used to prevent recurrent FS, and fever control medications should only be administered as needed to manage fever. Proper recognition of red flags can guide appropriate management and interventions, laying the groundwork for suitable follow-up. Finally, providing detailed counseling to the child’s family is essential to help them cope with the traumatic experience. We recommend scheduling a consultation with families within 2–3 weeks after the first convulsive event to assist them in coping with the traumatic experience.

## Data Availability

PubMed (https://pubmed.ncbi.nlm.nih.gov/) and Web of Science (https://access.clarivate.com/) databases.
